# Comparative Lipidomic and Metabolomic Analyses Reveal the Mystery of Lacquer Oil from *Toxicodendron vernicifluum* for the Treatment of “Yuezi” Disease in Nujiang, China: From Anti-Inflammation and Anti-Postpartum Depression Perspective

**DOI:** 10.3389/fphar.2022.914951

**Published:** 2022-06-13

**Authors:** Liya Liu, Fei Cai, Yitong Lu, Yuting Xie, Hao Li, Chunlin Long

**Affiliations:** ^1^ Key Laboratory of Ecology and Environment in Minority Areas (Minzu University of China), National Ethnic Affairs Commission, Beijing, China; ^2^ College of Life and Environmental Sciences, Minzu University of China, Beijing, China; ^3^ Key Laboratory of Ethnomedicine (Minzu University of China), Ministry of Education, Beijing, China

**Keywords:** lacquer oil, anti-infammation, anti-postpartum depression, lipidomics, metabolomics, “*yuezi*” disease, ethnopharmacology

## Abstract

**Background:** In southwest China, especially in Nujiang, lacquer oil from the drupes of *Toxicodendron vernicifluum* (Stokes) F. A. Barkley, including black lacquer oil (BLO) and white lacquer oil (WLO), is one of the most important edible oils for the local people. Through the field investigation, the locals believe that lacquer oil has benefits for parturient women and for the treatment of “Yuezi” disease. However, studies on bioactivities and the chemical compositions of lacquer oil are limited.

**Purpose:** This study was designed to reveal the mystery of lacquer oil for the treatment of “Yuezi” disease by testing its anti-inflammatory and anti-postpartum depressant activities and related bioactive compounds.

**Methods:** The anti-inflammatory effects of lacquer oil were examined by establishing a lipopolysaccharide (LPS)-induced RAW264.7 cell inflammation model and detecting the level of pro-inflammatory factors such as NO, IL-6 and TNF-α. The antidepressant effects of lacquer oil were studied by building a mouse model of postpartum depression (PPD), and the animal behavior changes of PPD model mice were assessed by open field test (OFT), forced swimming test (FST) and tail suspension test (TST). The chemical profiles of BLO and WLO were detected by lipidomic and the untargeted metabolomic research methods based on UPLC-MS/MS.

**Results:** The results showed that BLO and WLO exerted anti-inflammatory effects by reducing the release of pro-inflammatory factors and BLO had better anti-inflammatory effects than WLO. While only BLO had anti-postpartum depressant activities, as evidenced by the significantly reduced the immobility time of the BLO-treated PPD mice in TST and FST compared to the PPD model mice. The comparative lipidomic analysis revealed that BLO contained high levels of Diacylglycerols (DAG) and Diacylglyceryl trimethylhomoserines (DGTS) but low level of ceramides (Cer), sphingomyelines (SM), phosphatidylcholines (PC) and phosphatidylethanolamines (PE) compared with WLO. Metabolomics analysis showed that there were 57 chemical markers between BLO and WLO, of which 17 potential biomarkers have been declared to possess anti-inflammatory and/or antidepressant activities.

**Conclusion:** The findings of this study furnish a scientific support for the traditional uses of lacquer oil for the treatment of “Yuezi” disease from anti-inflammation and anti-postpartum depression perspective.

## 1 Introduction

In China, for over 2000 years, most parturient women are involved in a very ancient custom known as “Zuo Yuezi”, also referred to “confinement in childbirth” or “1 month post-natal care”. The traditional practice of “Zuo Yuezi” requires women to stay at home for a month after delivery, which is deemed to promote postpartum recovery, improve the future health, and prevent disease (Liu et al., 2015). If a new mother is not provided with the best care from herself or her family, she is in danger of developing “Yuezi” disease. “Yuezi” disease is a physical and/or psychological disorder of parturient women characterized by a series of symptoms, like hemorrhaging, venous thromboembolic events, arthralgia, breast issues, infection, postpartum anxiety and depression, obesity and undernutrition, which may be due to the poor cares during “Yuezi” period (Tripette et al., 2014; Mao et al., 2016; WHO et al., 2019). It has been demonstrated that approximately 60% of women in China reported at least one of these symptoms (Mao et al., 2016). While the Lisu and Nu ethnic women in Nujiang Prefecture of Yunnan Province do not require “Zuo Yuezi” for maternity. In addition to customs and other reasons, the Lisu and Nu parturient women have traditional health remedies to prevent and treat “Yuezi” disease.

*Toxicodendron vernicifluum* (Stokes) F. A. Barkley, commonly known as lacquer tree or sumac, is a deciduous tree species in the family Anacardiaceae, which is a species endemic to Asia (Editorial Committee of Flora of China, 1980). Lacquer trees are a special traditional resource in China, which not only secrete raw lacquer, but also contains rich oil in its drupes, the content of which is 45%–55%. In southwest China, particularly in Nujiang, lacquer trees have a long history of cultivation and use, and are distributed or cultivated all over the territory, especially in Fugong County and Lushui City (Long et al., 1999; Lin et al., 2002; Long et al., 2003; Hu and Long, 2007). As the branches, leaves, resins, drupes, seeds, and even just passing around the lacquer trees could cause allergies, people from other areas only use the lacquer resin for furniture paint, but the lacquer oil have been used as edible oil for thousands of years by minority people living in Nujiang and surrounding areas (Gao and Sha, 2015). According to our field investigation, lacquer oil is the principal edible oil of Lisu, Nu and Dulong people living in Nujiang (Zhou et al., 2011; Han and Cui, 2012; Gao and Sha, 2015; Zhang et al., 2017). In Nujiang, lacquer oil is also a typical “nutraceutical” food (Zhang et al., 2017). Local people believe that lacquer oil is beneficial to the postpartum recovery for parturient, that is lacquer oil could prevent and cure “Yuezi” disease (Gao and Sha, 2015). For example, Lisu or Nu parturient women have the habits of eating lacquer oil chicken, and drinking water mixed with lacquer oil and sugar, which are the most important tonics for them. They are able to go to work safely in the fields for only three or 4 days after delivery, and would not suffer from “Yuezi” disease in the future.

In Nujiang, lacquer oil is extracted by pressing the mesocarp of drupes, the fruits of lacquer tree, which are harvested from September to October each year (Wang, 2013). As its high melting point, the oil would turn into a waxy substance at room temperature, so it is also called “lacquer wax”. There are two different types of lacquer oil, black lacquer oil (BLO) and white lacquer oil (WLO). BLO is mostly brown or black solid, slightly different from WLO, which is mostly light yellow, gray or gray-green in color. BLO is made by pressing the mesocarp of immature fruit grown at higher altitudes, while WLO is made by pressing the mesocarp of mature fruits grown at lower altitudes. And the locals believe that BLO is of better quality and has better medicinal and nutritional values (Gao and Sha, 2015).

However, pharmacological effects of lacquer oil have not yet been firmed up by modern medicine. As postpartum infection and postpartum depression (PPD) are the two common events among mothers in “Yuezi” (Ahnfeldt-Mollerup et al., 2012; Ceriani Cernadas, 2020), the potential anti-inflammatory and anti-depressant activities of lacquer oil may be of critical importance to explain the mechanisms of lacquer oil on preventing and treating “Yuezi” disease. So, the first step of the present work was to explore the anti-inflammatory and anti-postpartum depression activities of BLO and WLO by building a lipopolysaccharide (LPS)-induced inflammation model in RAW264.7 cells and a postpartum depression (PPD) mice model, which will provide a partial experimental basis for the treatment of “Yuezi” disease by lacquer oil.

Besides, previous researches of lacquer oil mainly focused on the extraction and the compositional analysis of fatty acid. The analysis results of the higher fatty acid composition of lacquer oil showed that the higher fatty acids in lacquer oil from Nujiang were mainly saturated fatty acids such as palmitic acid, oleic acid, stearic acid, arachidic acid and eicosanodic acid, among which the content of palmitic acid is the highest, with an average content of about 60%. While the level of polyunsaturated fatty acids (PUFAs) with high nutritional and medicinal value, such as linolenic acid, linoleic acid and arachidonic acid, was low. The results also show that the fatty acid composition of BLO and WLO has negligible difference (Lin et al., 2002; Wang et al., 2019; Xie, 2019; Li et al., 2020). Therefore, the analysis of the fatty acid composition of lacquer oil fails to explain the pharmacodynamic differences between BLO and WLO. Thus, our group speculated that there are some protective secondary metabolites existed in the lacquer oil, such as vitamins, carotenoids, phytosterol, β-carotene, and phenolic compounds, which are responsible for its nutritional and medicinal effects. However, no related work has been published so far. Based on this hypothesis, the second step of the present work was to investigate the pharmacological material basis of lacquer oil by analyzing the composition of lipids and secondary metabolites of different lacquer oil products by using lipidomic and the untargeted metabolomics research methods based on ultra-performance liquid chromatography-integrated mass spectrometry (UPLC-MS). Our study has made it possible to gain a better knowledge of the traditional uses of lacquer oil for treatment of “Yuezi” disease in Nujiang.

## 2 Materials and Methods

### 2.1 Samples Collection

Lacquer oil samples, include black lacquer oil (BLO) and white lacquer oil (WLO), were collected from the local market in Lushui City, Nujiang Lisu Autonomous Prefecture, Yunnan Province, China. Traditional knowledge associated with BLO and WLO had been investigated, through semi-structured interview and participatory observation in local markets and villages, and informed consent was obtained verbally from all participants prior to the study.

### 2.2 Chemicals

LPS from *Escherichia coli* 0111:B4 (catalog No.: L4391) was obtained from Sigma-Aldrich (St. Louis, Missouri, United States). High-glucose Dulbecco’s modified Eagle’s medium (DMEM-H, catalog No.: 11965084), Fetal bovine serum (FBS, catalog No.: 10099141) and Penicilin-stretomycin solution (catalog No.: 11548876) were obtained from Gibco (New York, United States). 2, 2, 2-Tribromoethand (catalog No.: T903147) and tert-Amyl alcohol (catalog No.: A800283) were obtained from Shanghai Macklin Biochemical Co., Ltd (Shanghai, China). Progesterone (P4, catalog No.: S30586), Estradiol benzoate (EB, catalog No.: S30633) and Sesame oil were purchased from Shanghai Yuanye Bio-Technology Co., Ltd (Shanghai, China).

### 2.3 Anti-Inflammatory Activity Assay

#### 2.3.1 Cell Culture

RAW264.7 cells, obtained from Procell Life Science & Technology Co., Ltd. (Wuhan, China), were cultured in DMEM-H supplemented 10% FBS, 1% penicilin-stretomycin solution in a humidified incubator with 5% CO_2_ at 37°C.

#### 2.3.2 Cell Viability Test

The RAW264.7 cells (4 × 10^4^ cells/well) were cultured in 96-well plates for 12 h and then with BLO, WLO treatment (0–10 mg/ml) for 24 h. Thereafter, the cell viability was detected by using the Cell Counting Kit (CCK)-8 (Gen-view Scientific INC., Beijing, China; catalog No.: GK3607), and the experimental protocol was performed according to the manufacturer’s instructions.

#### 2.3.3 NO, IL-6 and TNF-α Assay

The RAW264.7 cells (8×10^5^ cells/well, seeded in 6-well plates for 12 h) were pretreated with BLO or WLO (0, 0.5 1, 2.5, 5 mg/ml) for 1 h, then added LPS (0.05 μg/ml) and incubated for another 18 h. Thereafter, the culture supernatant was used to measure the levels of NO, IL-6 and TNF-α. NO level was detected by using a Nitric oxide assay kit (Biorigin (Beijing) Inc., Beijing, China; catalog No.: BN27106) based on the manufacturer’s recommendations. The protein levels of IL-6 (catalog No.: SEA079Mu) and TNF-α (catalog No.: SEA133Mu) were tested by ELISA Kit (Cloud-Clone Corp., Wuhan, China) depending on the product protocols.

### 2.4 Anti-Postpartum Depression Activity Assay

#### 2.4.1 Animals

A total of 32 female C57BL/6 mice (weighing 14–18 g, aged 8 weeks, obtained from SPF (Beijing) Biotechnology Co., Ltd., Beijing, China) were feed under controlled light/dark cycle, temperature and humidity. They were provided free access to water and food before and after all procedures. The experimental protocol was approved by the Biological and Medical Ethics Committee of Minzu University of China (ECMUC2021007AO) and performed in accordance with the ethics guidelines.

#### 2.4.2 Postpartum Depression Mice Model Establishment

The PPD mice model was established by EB withdrawal after hormone-simulated pregnancy as the previous study described (Zhang et al., 2016). In brief, a skin incision of 0.5–1.0 cm in length was made along the posterior midline of the back. The skin of the incision was separated from the subcutaneous tissue to the left and right, and the skin incision was first stretched horizontally to the left about 0.5 cm to find the ovary, a milky bright cellulite, which was close to the lower pole of the kidney. The muscle layer on the surface of the fatty mass was lifted with forceps, and the muscle layer was cut longitudinally with ophthalmic scissors along the lateral border of the psoas major muscle, about 0.5 cm long, and the left ovary of the mouse could be found in this fatty mass. After ovariectomy, the cut muscle layer was repaired with a simple 5/0 silk suture, and the right ovary of the mouse could be removed in the same way. The cut muscle layer was repaired and the skin incision was finally closed with a needle of 5/0 silk thread.

Mice with bilateral ovariectomy (OVX mice) or sham ovariectomy were given 7 days to recover. On day 8 after operation, 0.1 ml of a mixture consisting of EB (0.5 μg/day) and P4 (0.8 mg/day) was injected subcutaneously into OVX mice at 16:30–17:00 for 16 days, and followed by EB (10 µg/day) for another 7 days. Sham ovariectomy mice were injected subcutaneously with 0.1 ml of sesame oil, which also was used to prepare EB and P4.

#### 2.4.3 Experimental Grouping and Drug Administration

According to the recommended lipid intake of Chinese people (25 g/d) recommended by Chinese Nutrition Society, the level of lipid supplementation in mice was 3.8% based on the energy ratio of lipid intake. The 32 mice were assigned into the control group (8, sham ovariectomy mice fed with normal diet containing 4% soybean oil for 30 days), the model group (8, PPD mice fed with normal diet containing 4% soybean oil for 30 days), the BLO group (8, PPD mice fed with the custom diet containing 3.8% BLO for 30 days), the WLO group (8, PPD mice fed with the custom diet with 3.8% WLO for 30 days). The diets were prepared as shown in Supplementary Table S1.

#### 2.4.4 Behavioral Examinations

##### 2.4.4.1 Open Field Test

The Open Field Test (OFT) is designed to evaluate the autonomous activities, exploratory activities, and degree of nervousness of experimental objects in situations that are unfamiliar to them. OFT was carried out in an OFT box (50 cm × 50 cm × 20 cm) as the previously described (Li et al., 2021a). The mice were positioned in the center of the bottom of the box and permitted to explore the apparatus for 5 min. The mice movements were automatically recorded and analyzed by ANY-maze software (Global Biotech, United States). The total distance and the time they spent in the central region were the parameters that were used to assess.

##### 2.4.4.2 Forced Swimming Test

The Forced Swimming Test (FST) is a validated method for detecting behavioral despair in depressed animal. The FST was performed as previously described (Jiang et al., 2018). Briefly, mice were forced to swim for 6 min, and monitored by a forced swimming analysis system (Chengdu Taimeng Software Co. Ltd., Sichuan, China). The swimming immobility time (i.e., the mouse only did a slight movement to keep its head above the water) in the last 4 min were recorded and analyzed.

##### 2.4.4.3 Tail Suspension Test

The Tail Suspension Test (TST) was also used to evaluate behavioral despair and carried out as previously described (Jiang et al., 2018). Briefly, the tail of each mouse was taped to the support of the suspension box and hung upside down with its head about 20 cm from the bottom of the box. The trials were conducted for 6 min, and the immobility time in the last 4 min was recorded and analyzed. When mice stopped struggling and kept completely motionless were considered to be immobile.

### 2.5 Lipidomic Study

100 mg of lacquer oil samples were added to a glass centrifuge tube with a Teflon-lined cap, and then added 0.75 ml of pre-cooled methanol and vortex shaked. 2.5 ml pre-cooled methyl tert-butyl ether was added and incubated on a shaker at room temperature for 1 h. 0.625 ml MS-grade water was added and mixed to stratify the organic phase. After incubation at room temperature for 10 min, the organic phase was centrifuged at 1000 g for 10 min. The upper organic phase was collected, and 1 ml mixed solvent (methyl tert-butyl ether/methanol/water (10:3:2.5, v/v/v) was added to the lower layer (water and methanol) to extract the upper organic phase again. The organic phase collected twice was concentrated by nitrogen blowing with a nitrogen blower, and then redissolved with 100 μL isopropyl alcohol, and later detected by LC-MS/MS system. This procedure was performed six times (*n* = 6) for both BLO and WLO.

The lipidomic profiling was performed using a Vanquish Ultra-High Performance Liquid Chromatography (UHPLC) system (Thermo Fisher, Germany) coupled with an Orbitrap Q Exactive™ HF mass spectrometer (Thermo Fisher, Germany) at Beijing Novogene Technology Co., Ltd (Beijing, China) as previous described (Xu et al., 2022).

### 2.6 Untargeted Metabolomics Study

100 mg of lacquer oil samples grounded by liquid nitrogen were placed into EP tubes and added 500 μL of 80% methanol aqueous solution. Then vortex oscillation, ice bath for 5 min, 15,000 g, 4°C centrifugation for 20 min. A certain amount of supernatants were diluted with MS grade water to a final concentration containing 53% methanol. The samples were subsequently centrifuged at 15,000 g for 10 min at 4°C, and the supernatants were subsequently collected and analyzed by LC-MS/MS. This procedure was performed six times (*n* = 6) for both BLO and WLO.

UHPLC-MS/MS analyses were performed using a Vanquish UHPLC system (Thermo Fisher, Germany) coupled with an Orbitrap Q Exactive™ HF-X mass spectrometer (Thermo Fisher, Germany) at Beijing Novogene Technology Co., Ltd (Beijing, China) as previous described (Yu et al., 2022).

### 2.7 Statistical Analysis

In the bioactivities assays, the results were expressed as means ± standard error of mean (means ± SEM) values of at least three replicates unless otherwise stated. All statistical analyses were carried out using GraphPad Prism 9.3.1 software (GraphPad Software, San Diego, United States). The normality test indicated that the data were normally distributed and met the criteria for using parametric tests, one-way ANOVA followed by a post hoc Tukey test or an unpaired parametric *t*-test, including Welch’s correction, was carried out. Values of *p* < 0.05 was regarded as significant.

For chemical profiles analysis, data were presented as the mean ± SEM of at least six replicates, and statistical analyses were performed using the statistical software R (R version R-3.4.3), Python (Python 2.7.6 version) and CentOS (CentOS release 6.6). PCA and OPLS-DA were performed at metaX. Univariate analysis (*t*-test) was used to calculate the statistical significance (*p*-value).

## 3 Results

### 3.1 Anti-inflammatory Effects of Lacquer Oil in Lipopolysaccharide-Induced RAW264.7 Cells

**FIGURE 1:**
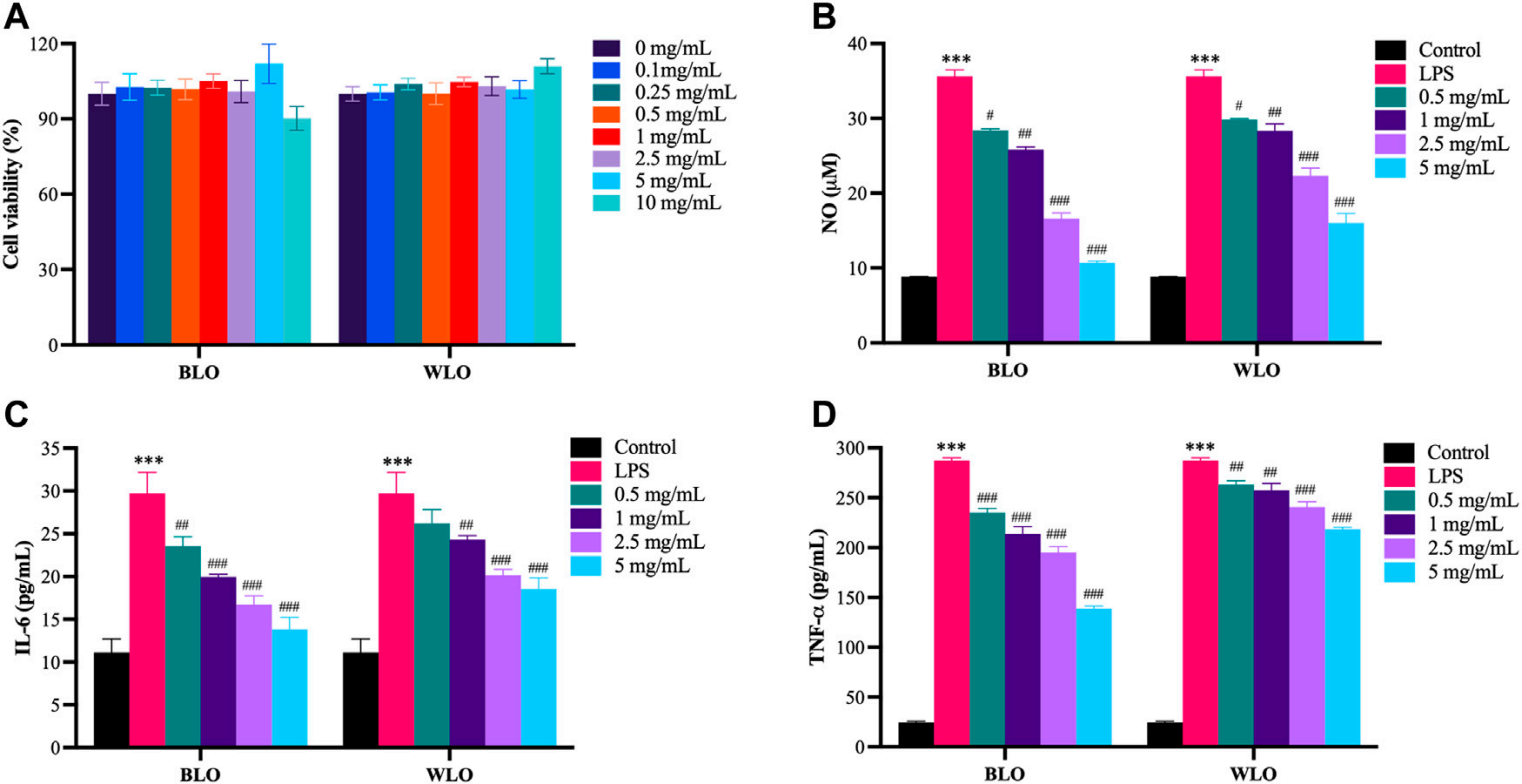
Anti-inflammatory effects of BLO and WLO in LPS-induced RAW264.7 cells (X ± SEM, *n* = 3). **(A)**: Cytotoxic effects of BLO and WLO on RAW264.7 cells. The cells were incubated with different concentrations of BLO and WLO for 24 h, and then the cell viability was detected by CCK-8 assay. **(B–D)**: Effects of BLO and WLO on NO **(B)**, IL-6 **(C)** and TNF-α **(D)** release in LPS-challenged RAW264.7 cells. The RAW264.7 cells were treated with BLO or WLO for 1 h and then added LPS for an additional 18 h ***p < 0.001 vs. Control group. ^#^ *p* < 0.05, ^##^ *p* < 0.01, ^###^ *p* < 0.001 vs. LPS group.

We firstly evaluated the cytotoxic effects of lacquer oil in RAW264.7 cells. The cells were treated with different concentration of BLO or WLO (0.1, 0.25, 0.5, 1, 2.5, 5 and 10 mg/ml) for 24 h, and then the cell viability was determined by the CCK-8 assay. The results indicated that both BLO and WLO had no obvious cytotoxic effects on RAW264.7 cells up to 10 mg/ml for 24 h (Figure 1A).

Furthermore, to explore the anti-inflammatory effects of lacquer oil, RAW264.7 cells challenged with LPS was employed to mimic chronic inflammation. As shown in Figures 1B–D, LPS stimulation for 18 h significantly increased the production of pro-inflammatory factors such as NO, IL-6, and TNF-α in the supernatants, while BLO and WLO treatments markedly lowered these cytokine secretions in a concentration-dependent manner. Besides, compared to WLO, BLO had better anti-inflammatory effects in high concentrations (2.5 and 5 mg/ml) (Figures 1B–D).

### 3.2 The Effects of Lacquer Oil on the Depression-Related Behaviors in Postpartum Depression Model Mice

**FIGURE 2:**
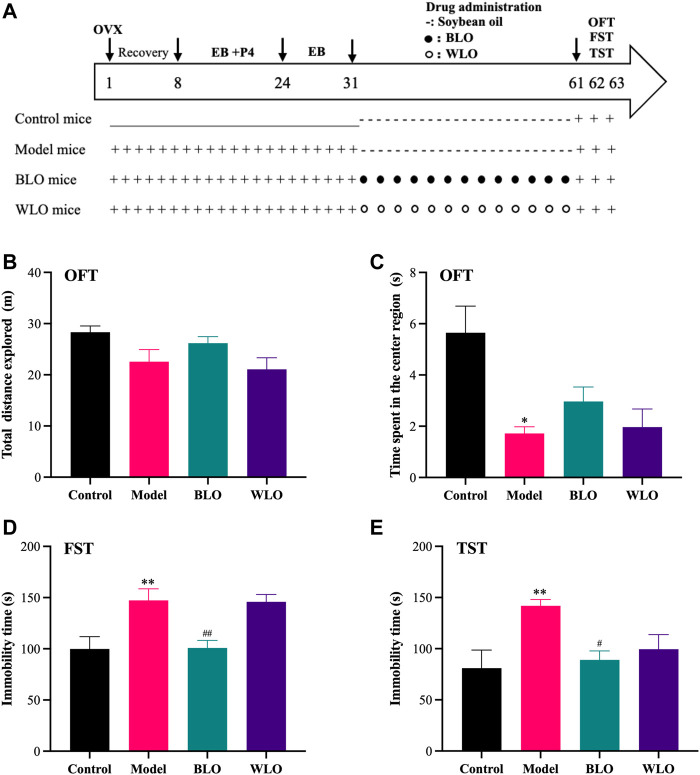
The effects of BLO and WLO on the depression-related behaviors in PPD model mice (X ± SEM, *n* = 8). **(A)**: Time course of the experimental procedure. **(B,C)**: The total distances explored and the time spent in the center region in OFT. **(D,E)**: The immobility time in FST and TST. *p < 0.05 vs. Control group; ^*#*^ *p* < 0.05, ^##^ *p* < 0.01 vs. Model group.

To estimate the effects of BLO and WLO on the locomotor behaviors of PPD model mice, we performed an OFT test, as shown in Figures 2A,B. From Figure 2A, we can see there is no significant different in the distances traveled by each group of mice (Figure 2A). Despite the fact that PPD mice (including the model group, the BLO group and the WLO group) showed a tendency to spend less time in the center region, the groups were unable to reach significance compared to the control group. (Figure 2B). Furthermore, as shown in Figure 2B, compared to the model group, there was no obvious difference in the time spent in the central region between the BLO group and the WLO group.

To evaluate the depression-like behaviors of the tested mice, we further carried out FST and TST tests, as shown in Figures 2C,D. In both of these tests, there was a significant increase in immobility time in the PPD model mice compared to the control mice, indicating that the PPD mice model was established successfully. Compared with the model group, BLO markedly decreased the immobility time of PPD mice in FST and TST, indicating BLO have effective antidepressant effects. However, WLO could also reverse the behavior changes of PDD mice, especially in the TST, but those changes were not significant compared to the model group.

### 3.3 Comparative Lipidomic Analysis Revealed the Different Lipid Constituents in Black Lacquer Oil and White Lacquer Oil

With a great difference in therapeutic effects of BLO and WLO, the chemical differences between BLO and WLO should be investigated. We first performed a highly sensitive and specific quantitative analysis of lipids in lacquer oil using UPLC-MS/MS to derive a comprehensive lipid composition profile.

**FIGURE 3:**
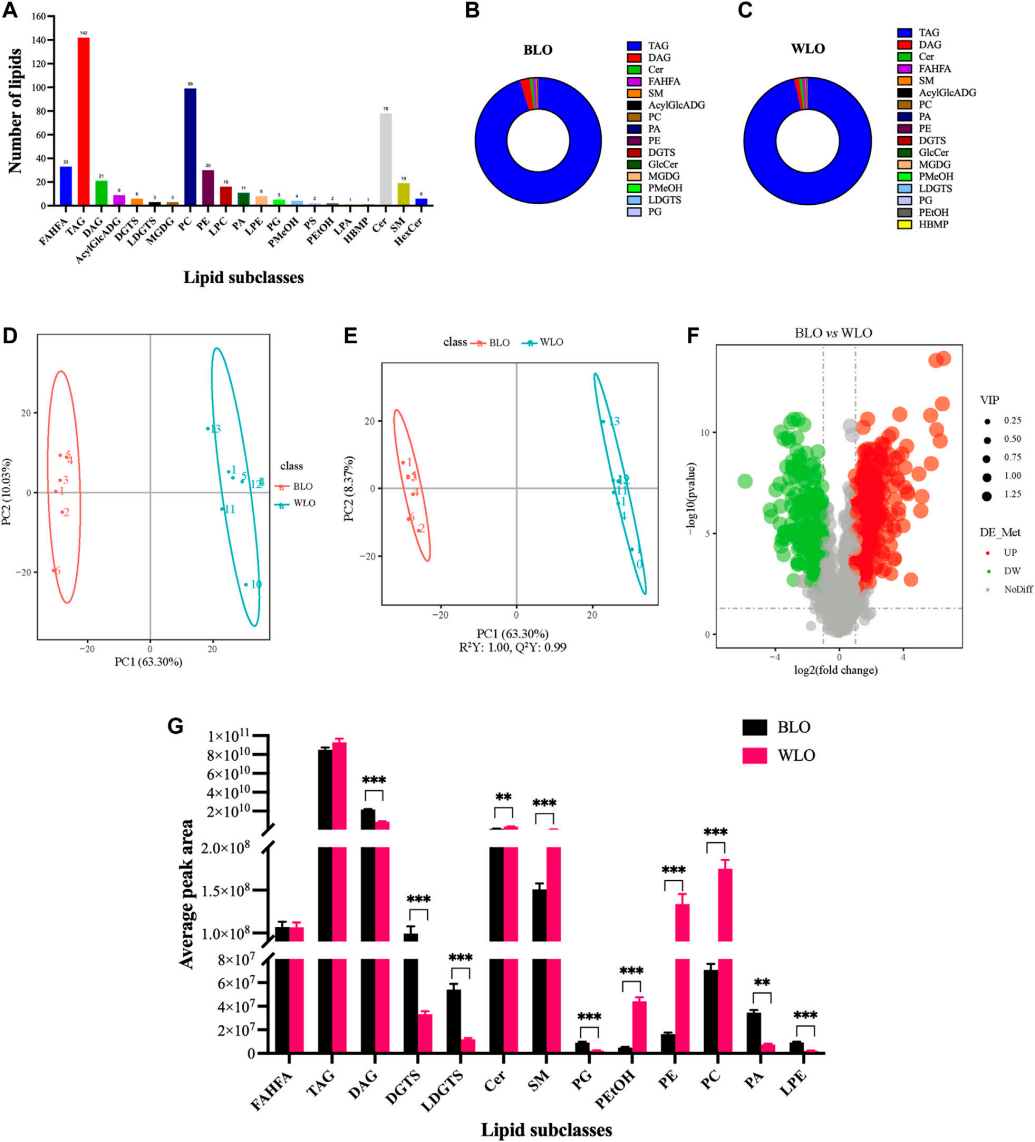
Lipidomic studies by UPLC-MS/MS on the lipid constituents of BLO and WLO. **(A)**: The number of lipids detected and annotated in BLO and WLO. **(B,C)**: The composition of lipids detected and annotated in BLO and WLO. **(D,E)**: Multivariate data analysis of UPLC-MS/MS data of BLO and WLO. Score scatter plot of the PCA **(D)** and OPLS-DA **(E)** derived from the UPLC-MS/MS data of BLO and WLO lipidomic profiles. **(F)**: Volcano plot showing the different lipids between BLO and WLO. **(G)**: Differences in the significantly different lipid subclasses between BLO and WLO. **p < 0.01, ***p < 0.001.

In the present study, the untargeted analysis of lipids from the two lacquer oil samples yielded 1305 features, of which 498 lipids were annotated. These lipids belonged to the four main lipid classes: fatty acyls (FA), glycerolipids (GL), glycerophospholipids (GPL) and sphingolipids (SP) (Figure 3A) and comprised of 33 fatty acid ester of hydroxyl fatty acids (FAHFA), 142 Triacylglycerols (TAG), 21 Diacylglycerols (DAG), 9 Acylglucuronosyldiacylglycerol (AcylGlcADG), 6 Diacylglyceryl trimethylhomoserines (DGTS), 3 Lysodiacylglyceryl trimethylhomoserines (LDGTS), 3 Monogalactosylmonoacylglycerols (MGDG), 99 Phosphatidylcholines (PC), 38 Phosphatidylethanolamines (PE), 16 Lysophosphatidylcholines (LPC), 11 Phosphatidic acids (PA), 8 Lysophosphatidylethanolamines (LPE), 5 Phosphatidylglycerols (PG), 4 Phosphatidylmethanol (PMeOH), 2 Phosphatidylserines (PS), 2 Phosphatidylethanol (PEtOH), 1 Lysophosphatidic acids (LPA), 1 Hemibismonoacylglycerophosphate (HBMP), 78 Ceramides (Cer), 19 Sphingomyelines (SM), 6 Hexsolyceramides (HexCer). The total ion chromatogram (TIC) and the performances of experimental QCs in the lipidomic experiments are provided in Supplementary Figures S1A–C. Of note, the acylglycerols TAG and DAG were the major class of lipids in lacquer oil (Figures 3B,C).

The side chain of lipids in lacquer oil have 30–60 carbon atoms and 0–12 double bonds, except for Lyso-lipids, whose fatty acid side chains have 14–22 carbon atoms and 0–6 double bonds; almost every lipid contains at least one fatty acid side chain with C16 or C18 and 0–3 double bonds. In addition, taking glycerides, the main component of lacquer oil, as an example, with higher sensitivity compared to gas chromatography, UPLC-MS/MS successfully identified a variety of beneficial PUFAs: Linoleic acid (C18:2), Linolenic acid (C18:3), Arachidonic acid (C20:4), Clupanodonic acid (C22:5), and Docosahexaenoic acid (C22:6).

In order to figure out how BLO is different from WLO, and which lipids contribute the most to this difference, multivariate statistical analysis was applied to the 498 lipids. Principal Component Analysis (PCA) and Orthogonal Projections to Latent Structures Discriminant Analysis (OPLS-DA), useful tools for comparing and identifying complex samples, were further performed to visualize the discrimination between analyze the differences of lipids in BLO and WLO. In the PCA model, the PC1 and PC2 accounted for 63.30% and 10.03% of the variance, respectively (Figure 3D). The results revealed a clear separation between BLO and WLO. In OPLS-DA analysis (Figure 3E), samples from BLO and WLO were completely separated, further suggesting that the lipid composition of BLO and WLO is significantly different.

Lipid selections based on Variable Importance in the Projection (VIP) from OPLS-DA, Fold Change (FC) and *p*-value from t-test were useful methods to identify markedly different compounds that help to distinguish between BLO and WLO. Finally, 109 lipids (VIP > 1, FC > 2.0 or FC < 0.5, *p*-value < 0.05) were remarkably different when comparing BLO and WLO, including 53 upregulated lipids and 56 downregulated lipids in black lacquer oil (Figure 3F). All significantly different lipids can be found in Supplementary Table S2.

Further analysis revealed that 13 lipid subclasses, including 6 upregulated lipid subclasses (DAG, DGTS, LDGTS, PA, PG and LPE) and 5 downregulated lipid subclasses (Cer, SM PC, PE, PEtOH,) were significantly different in BLO compared with WLO (Figure 3G). Interestingly, DAG, DGTS, Cer, SM, PC and PE changed robustly in the two groups. BLO contains a higher level of DAG and DGTS than that of WLO. The top significantly upregulated DAG and DGTS in BLO were DAG (20:0/20:4) and DGTS (16:0/16:1), respectively. While WLO contains a higher level of Cer, SM, PC and PE than that of BLO. The top significantly upregulated Cer, SM, PC and PE in WLO include Cer-BDS (d22:0/16:1), SM [d18:1/14:1 (9Z) (OH)], PC (19:0/19:0) and PE (16:0/22:1).

### 3.4 Chemical Profiling Revealed the Difference of Secondary Metabolites Composition Between Black Lacquer Oil and White Lacquer Oil

Next, we used an untargeted metabolomics approach based on UPLC-MS/MS to evaluate the heterogeneity of secondary metabolite between BLO and WLO. After normalization, in total, 910 compounds with known structures were identified, including carbohydrates, amino acids, fatty acids, carboxylic acid, organic acids, terpenoids, alkaloids, lignans, hormones, phenanthrenes, benzoyls, benzoic acids, phenylpropanoids, mycotoxins, polyphenols, sugars, etc. To our knowledge, this is the first comprehensive metabolite analysis of lacquer oil as a nutritional product that help provide material basis for their benefits on treatment of “Yuezi” disease. The total ion chromatogram (TIC) and the performances of experimental QCs in the untargeted metabolomics experiments are visualized in Supplementary Figures S2A–C.

**FIGURE 4:**
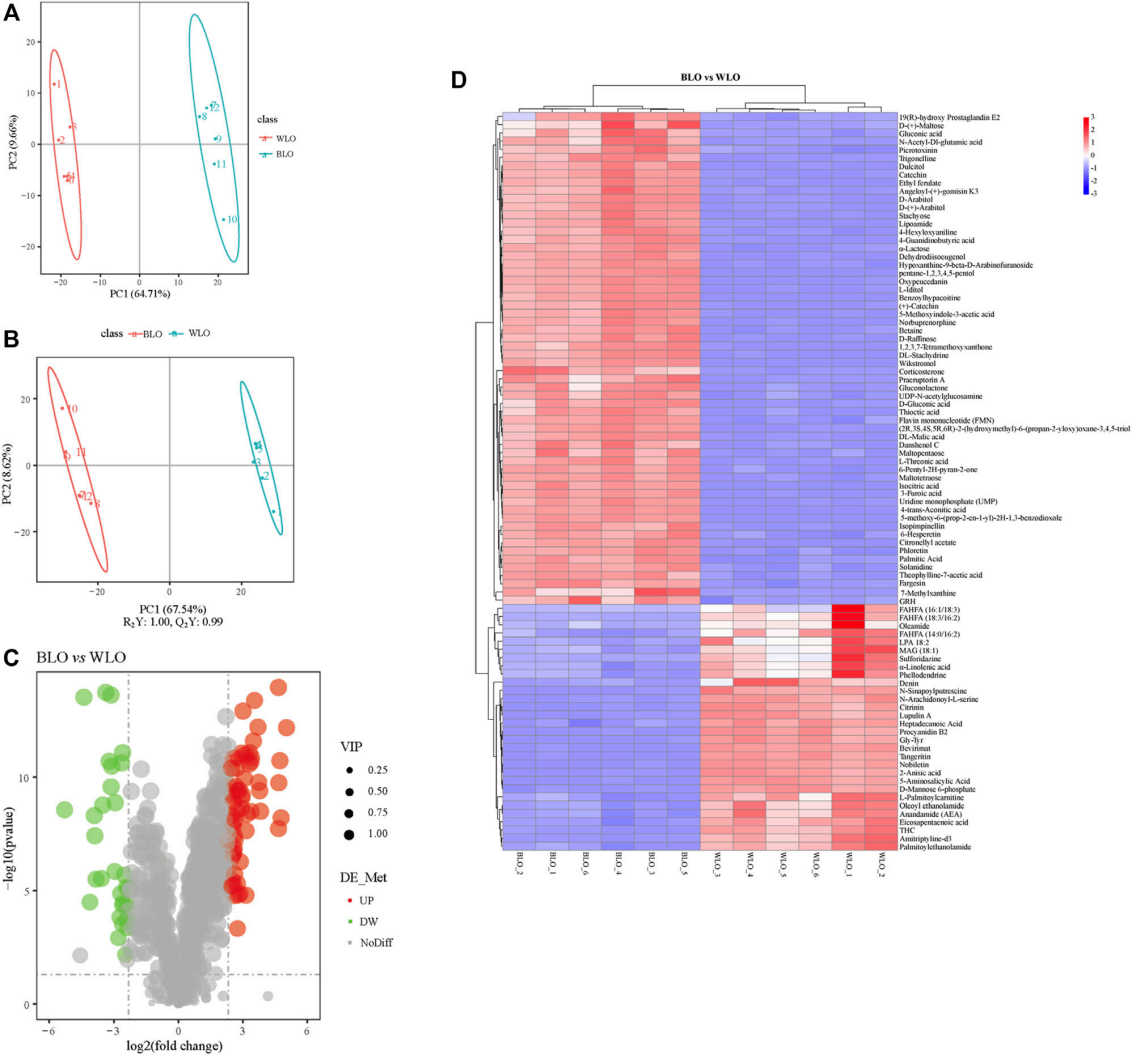
Untargeted metabolomics studies by UPLC-MS/MS on the secondary metabolites composition of BLO and WLO. **(A,B)**: Multivariate data analysis of UHPLC-MS data of BLO and WLO. PCA **(A)** and OPLS-DA **(B)** score plots derived from the UPLC-MS/MS data of BLO and WLO metabolite profiles. Volcano plot **(C)** and Heatmap **(D)** used to visualize the different metabolites in BLO compared to WLO.

In order to understand the differences between BLO and WLO in the composition of secondary metabolites, and which metabolites contribute the most to this difference, PCA was firstly performed on the 910 metabolites, as shown in Figure 4A. The PCA plot data showed that BLO and WLO are clearly separated, indicating the two groups exist significant differences in the composition of secondary metabolites. To further identify the chemical markers responsible for this separation, we performed OPLS-DA tests (Figure 4B). As the screening criteria, we used VIP > 1.0, FC > 5.0 and *p* < 0.05 to obtain the different metabolites between BLO and WLO. 57 chemical markers in BLO were identified and presented in Supplementary Table S3. And the results of screening differential metabolites were also visualized with a volcano plot and a heatmap (Figures 4C,D).

## 4 Discussion

In China, in addition to producing lacquer, the lacquer trees have long been used as a traditional herbal medicine for a variety of diseases, including infectious diseases, stomach disorders, blood disorders, and liver disease (Saravanakumar et al., 2019). The leaves, buds, flowers, bark, roots, seeds, and lacquer sap of the lacquer tree can be used as medicine, and their extracts have strong antioxidant, anti-inflammatory, anti-rheumatic, anti-tumor, anti-viral effects (Li et al., 2021b). At present, there are more studies on the medicinal use of lacquer leaves, roots and bark, while few studies have been conducted on the medicinal value of lacquer oil, a lacquer product that is closely related to the local people’s health and livelihood in Nujiang. In this study, our results showed that lacquer oil had good anti-inflammatory and anti-postpartum depression activities. In particular, BLO had better pharmacological activity than WLO, which may be attributed to the higher content of bioactive ingredients with anti-inflammatory and anti-depressant activities in BLO.

Postpartum infection is an infection that occurs after delivery. Up to a quarter of women will experience some infectious problem after childbirth. Breast infections are the most common, followed by wounds, airways, vaginal and urinary tract infections, endometritis and other infections (Ahnfeldt-Mollerup et al., 2012). These infections would cause various inflammatory diseases, such as mastitis, wound inflammation, respiratory tract inflammation, vaginal inflammation, urinary tract inflammation, etc., which is one of the common symptoms of “Yuezi” disease. Therefore, effective reduction of postpartum infection and inflammation is a key measure to promote rapid maternal recovery.

Here, in order to examine the anti-inflammatory activity of lacquer oil, we established a model of LPS-induced inflammation in RAW264.7 cells. When challenged with LPS, RAW 264.7 cells are activated and overproduce a variety of pro-inflammatory cytokines that lead to extensive histopathology and tissue damage (Dobrovolskaia and Vogel, 2002; Maldonado et al., 2016). While high concentrations of BLO and WLO used in this study strongly inhibited the LPS-induced release of NO, IL-6 and TNF-α, and BLO exhibited a better *in vitro* anti-inflammatory activity than WLO. In addition, CCK-8 assay showed no significant cytotoxic effect for the doses of LPS, BLO and WLO used in this study, indicating that anti-inflammatory effects of BLO and WLO were not a result of poor cell viability. These results suggested BLO could play an anti-inflammatory role in treatment of “Yuezi” disease by reducing the secretion of pro-inflammatory factors.

However, which compositions in BLO and WLO plays a major role in the anti-inflammatory effects remains elusive. Since BLO had a better *in vitro* anti-inflammatory activity than WLO, it was feasible to perform a stoichiometric modeling to identify marker compounds. The comparative lipidomic analysis revealed that BLO contains high level of DAG and DGTS but low level of Cer, SM, PC and PE. DAG is one of the major lipid subclasses in life-system and is a second messenger for a variety of cellular activities, and possesses a variety of beneficial health effects. Food with high in DAG has been reported to inhibit the accumulation of body fat and lower postprandial serum TAG, cholesterol and glucose levels (Tada and Yoshida, 2003; Lee et al., 2020). Besides, DAG has also been showed to improve bone health by increasing bone mineral density and improving bone microstructure (Choi et al., 2015). So, the high level of DAG, such as DAG (20:0/20:4) in BLO may be linked to halt the rise in postpartum obesity, cardiovascular disease and postpartum osteoporosis. Nonetheless, DAG has not been reported to have anti-inflammatory activity so far. DGTS is a phosphatidylcholine-free betaine lipid analogue. Acting as a carrier of acyl groups in the desaturation of fatty acid is the only known function of DGTS (Sato, 1992), and few biological studies have been conducted on DGTS to date. The only reported function of DGTS was that six DGTS discovered in the marine microalga *Nannochloropsis granulata* have anti-inflammatory activity (Banskota et al., 2013). Whether the DGTS contained in BLO also has anti-inflammatory activity requires further experimental study. In addition, previous studies have also reported low levels of PUFA, especially omega-3 fatty acids, in lacquer oil, while omega-3 fatty acids were shown to have anti-inflammatory effects (Calviello et al., 2013). Thus, lipids in BLO might not be involved in anti-inflammatory effects of lacquer oil.

Yet, a variety of protective chemical markers in BLO were identified by metabolomic analysis, and many of which have been reported to have anti-inflammatory properties, including 4 alkaloids, 3 coumarins, 3 lignans, 2 flavonoids, 2 phenylpropanoids, and 3 others. Among the 4 identified alkaloids, a series of inflammatory models have been applied to explore the anti-inflammatory activities and mechanisms of stachydrine and betaine. Intriguingly, stachydrine could effectively inhibit LPS-induced inflammatory bone loss by suppressing NF-κB and Akt signal pathway (Meng et al., 2019), suggesting its future use in the treatment of postpartum osteoporosis. Peng and his coworkers reviewed the anti-inflammatory mechanism of betaine, that is, betaine improves sulfur amino acid metabolism against oxidative stress, suppresses NF-κB activity and NOD-like receptor protein 3 (NLRP3) inflammasome activation (Zhao et al., 2018). Trigonelline is also a plant alkaloid with antioxidant, anti-inflammatory, and neuroprotective effects, which could reduce LPS-induced cognitive impairment and inflammatory response by regulating NF-κB/Toll like receptor 4 and acetylcholinesterase activity (Chowdhury et al., 2018; Khalili et al., 2018). Solanidine has been reported to reduce the level of interleukin-2 and interleukin-8 in Con A-induced Jurkat cells and NO production in LPS-challenged RAW264.7 cells (Kenny et al., 2013). Oxypeucedanin, a coumarin aglycone, can inhibit inflammatory reactions, such as inhibition of LPS-induced NO secretion in mouse peritoneal macrophages with IC_50_ values of 57 μM (Matsuda et al., 2005) and inhibition the secretion of TNF-α, interleukin-1β and interleukin-4 in DNP-HAS induced-RBL-2H3 cells allergic inflammation (Li and Wu, 2017). Robertson et al (2016) investigate the anti-inflammatory and pro-resolution activities of isopimpinellin using zebrafish neutrophilic inflammation model. They found that isopimpinellin reduced neutrophil migration towards tissue injury by PI3K inhibition to limit the further neutrophils recruitment to areas of inflammation (Robertson et al., 2016). Another coumarin in BLO, praeruptorin A may plays an anti-inflammatory role in RAW 264.7 cells by inhibiting of LPS-stimulated NF-κB pathway (Yu et al., 2011). There are 3 lignans in BLO reported to have the anti-inflammatory effects. Dehydrodiisoeugenol has been shown to suppress activation of NF-κB and expression of cyclooxygenase-2 in LPS-stimulated RAW264.7 cells (Murakami et al., 2005). Laavola et al (2017) examined the anti-inflammatory effects of nortrachelogenin in Murine J774 macrophages and in carrageenan-induced paw inflammation in mice, which were mediated by inhibiting the production of inflammatory factors (Laavola et al., 2017). Fargesin exhibits anti-inflammatory effects on phorbal ester-stimulated THP-1 human monocytes and LPS-stimulated RAW264.7 macrophages *via* suppression of NF-κB activation (Pham et al., 2017; Yue et al., 2018). Hesperetin, a flavonoid, could suppress MAPK and TLR4/NF-κB signaling to alleviate LPS-induced neuroinflammation (Jo et al., 2019; Muhammad et al., 2019). Phloretin is also a flavonoid with promising anti-inflammatory effects, and many models of inflammation have also been used to explore the anti-inflammatory activity and mechanism of phloretin (de Oliveira, 2016). Sunil et al (2021) reported that the protective effects of (+)–catechin against RAW 264.7 cells inflammation induced by LPS *via* inhibiting the expression of NF-κB and p38 MAPK (Sunil et al., 2021). Ethyl ferulate and myristicin are phenylpropanoid with well-documented anti-inflammatory effects. Ethyl ferulate, a derivative of ferulic acid, has been shown to inhibit RAW 264.7 cells inflammation induced by LPS and protect against acute lung injury induced by LPS *via* activation of nuclear erythroid 2- related factor (Wang et al., 2021; Wu et al., 2021). Lee and Park, 2011 evaluated the anti-inflammatory effects of myristicin on RAW264.7 cells stimulated by double-stranded RNA (dsRNA), found that it could inhibit NO, cytokines, chemokines and growth factors in dsRNA-stimulated RAW264.7 cells *via* the calcium pathway (Lee and Park, 2011). In addition, DL-malic acid could reduce 2, 4-dinitrochlorobenzene-induced inflammatory responses in atopic dermatitis-like skin lesions *in vitro* and *in vivo*, and improve skin conditions of AD mice (Lee et al., 2019). And thioctic acid exhibited an anti-inflammatory effect through inhibition of NF-κB, TNF-α and IL-6 and enhancing anti-inflammatory proteins, such as NF-E2-related factor-2 (Moura et al., 2015; Tibullo et al., 2017). In all, seventeen of the 57 chemical markers in BLO in Supplementary Table S3 have been reported to exhibited anti-inflammatory effects, indicating that these compounds are the main active components of the anti-inflammatory effects of lacquer oil, and BLO has a prominent anti-inflammatory potential that can provide protection against inflammatory diseases related “Yuezi” disease.

Inflammation is also engaged in the pathological processes of various “Yuezi” diseases, such as pain (Matsuda et al., 2019), and postpartum depression (Payne and Maguire, 2019). And the last is the most common complication during childbirth and occurs in about 10%–20% of women after childbirth, and depression-related suicides make up about 20% of postpartum death (Payne and Maguire, 2019; Ceriani Cernadas, 2020). Moreover, maternal depression adversely affects the behavioral, emotional, and cognitive development of the infant. Thus, we have established a PPD model in mice to study the antidepressant effect of lacquer oil. The FST and TST conducted in this study showed that supplementation with BLO for 30 days was effective and reversed the depression-like behaviors changes in PPD mice. Moreover, OFT results showed that the autonomous motor abilities of PDD mice were not affected in all the groups, indicating that the antidepressant effects of BLO were not attributable to the locomotive activity. So, BLO is a potential supplement in managing the development of postpartum depression.

Numerous studies have shown that fish oil supplementation in a PPD rat model could exert antidepressant-like effects, possibly because it is rich in omega-3 fatty acids (Burhani and Rasenick, 2017; Abdul Aziz et al., 2020). While our and previous studies have shown that omega-3 fatty acids are relatively low in BLO, so some protective micronutrients in BLO may be responsible for its antidepressant-like activities. Literature reviews revealed that of 17 bioactive compounds mentioned above, 5 compounds also had antidepressant effects, namely betaine, trigonelline, thioctic acid, praeruptorin A, hesperetin. Betaine markedly cut the immobility time in the FST and the latency to feed in the novelty suppressed feeding test, suggesting a possible antidepressant activity, and it also ameliorated the antidepressant effects of S-adenosyl-methionine and ketamine (Kim et al., 2013; Hao et al., 2019; Ubaldo-Suarez et al., 2019). Trigonelline is a pyridine alkaloid with antidepressant- and anxiolytic-like effects, as evidenced by its ability to significantly reduce immobility time in the FST *via* inhibiting N-methyl-Daspartate receptor activity and increasing hippocampal CA1 region. It reverses the negative effects of maternal separation on the behavior that was accompanied by reducing oxidative stress as well as increasing antioxidant capacity (Di Pierro et al., 2015; Lin et al., 2016). Thioctic acid has been reported to augment the antidepressant-like effects of antidepressants, such as desvenlafaxine and mirtazapine, on corticosterone-induced depression model (Oliveira et al., 2017; de Sousa et al., 2018). Praeruptorin A has been shown to improve depressive behaviors induced by chronic unpredicted mildly stressed in rats, and the mechanism may be related to improved synaptic ultrastructure and increased neurotrophic factors and nerve growth factors in the hippocampal CA1 region (Wang and Xu, 2014). Liu et al. (2015) reported that hesperetin improved depression and anxiety -like behaviors in diabetic rats *via* activation of Nrf2/ARE pathway. In addition, among 57 chemical markers of BLO, there are also 6 compounds with neuroprotective effects, which may also play a role in the anti-depression of BLO, namely sopimpinellin (Hao et al., 2019), DL-stachydrine (Cheng et al., 2020), lipoamide (Hou et al., 2019), ethyl ferulate (Scapagnini et al., 2004; Cunha et al., 2019), (+)-catechin (Bernatoniene and Kopustinskiene, 2018; Pervin et al., 2019), and myristicin (Ha et al., 2020). In all, eleven of 57 chemical makers have been reported to exhibit antidepressant effect or neuroprotective effects, indicating that these compounds are the main active components of the antidepressant effect in BLO.

## 5 Conclusion

To sum up, the anti-inflammatory and antidepressant effects of BLO and WLO were investigated and chemical markers were also identified by UPLC-MS/MS. In the present study, BLO has better anti-inflammatory and anti-postpartum depressant activities than WLO. OPLS-DA comparisons between BLO and WLO revealed 53 lipids and 57 chemical markers may contribute to the different therapeutic effects of BLO. Of all, 17 potential biomarkers have been reported to possess anti-inflammatory and/or antidepressant potential. Therefore, the bioactivities of these 17 biomarkers may furnish a scientific support for the traditional uses of lacquer oil from Nujiang for treatment of “Yuezi” disease from anti-inflammation and anti-postpartum depression perspective.

## Data Availability

The original contributions presented in the study are included in the article/Supplementary Material, further inquiries can be directed to the corresponding author.

## Glossary

AcylGlcADG: Acylglucuronosyldiacylglycerol
Akt: Protein kinase B
BLO: Black lacquer oil
Cer: Ceramides
DAG: Diacylglycerols
DGTS: Diacylglyceryl trimethylhomoserines
EB: Estradiol benzoate
FST: Forced swimming test
HBMP: Hemibismonoacylglycerophosphate
HexCer: Hexsolyceramides
LDGTS: Lysodiacylglyceryl trimethylhomoserines
IL-6: Interleukin-6
LPA: Lysophosphatidic acids
LPC: Lysophosphatidylcholines
LPE: Lysophosphatidylethanolamines
LPS: Lipopolysaccharide
NF-κB: Nuclear factor kappa B
NO: Nitric oxide
MAPK: Mitogen-activated protein kinase
MGDG: Monogalactosylmonoacylglycerols
OFT: Open field test
OPLS-DA: Orthogonal projections to latent structures discriminant analysis
P4: Progesterone
PA: Phosphatidic acids
PC: Phosphatidylcholines
PCA: Principal component analysis
PE: Phosphatidylethanolamines
PEtOH: Phosphatidylethanol
PI3K: Phosphatidylinositol 3 kinase
PG: Phosphatidylglycerols
PMeOH: Phosphatidylmethanol
PPD: Postpartum depression
PS: Phosphatidylserines
SM: Sphingomyelines
TAG: Triacylglycerols
TNF-α: Tumor necrosis factor-α
TST: Tail suspension test
UPLC-MS: Ultra-performance liquid chromatography-integrated mass spectrometry
WLO: White lacquer oil
