# Supplementary material for: Comparative Lipidomic and Metabolomic Analyses Reveal the Mystery of Lacquer Oil from Toxicodendron vernicifluum for the Treatment of “Yuezi” Disease in Nujiang, China: From Anti-Inflammation and Anti-Postpartum Depression Perspective
Source: Front Pharmacol. 2022 Jun 13;13:914951. doi: 10.3389/fphar.2022.914951 (PMC9234167; doi:10.3389/fphar.2022.914951)
Supplement: Supplementary file 1 [file DataSheet1.pdf]

Supplementary Materials

1 Supplementary Table S1

SUPPLEMENTARY TABLE S1 The composition of the diets (%)

Ingredient	Control group	Model group	WLO group	BLO group
Corn starch	55	55	55	55
Wheat bran	10.2	10.2	10.2	10.2
Soybean meal	17	17	17	17
Fish meal	8	8	8	8
Beer yeast	2	2	2	2
Premix	1	1	1	1
Soybean oil	4	4	0.2	0.2
WLO	0	0	3.8	0
BLO	0	0	0	3.8

The composition of diets was formulated based on Laboratory Animals Nutrients for Formula Feeds of China [GB 14924.3-2010].

2 Supplementary Figure S1

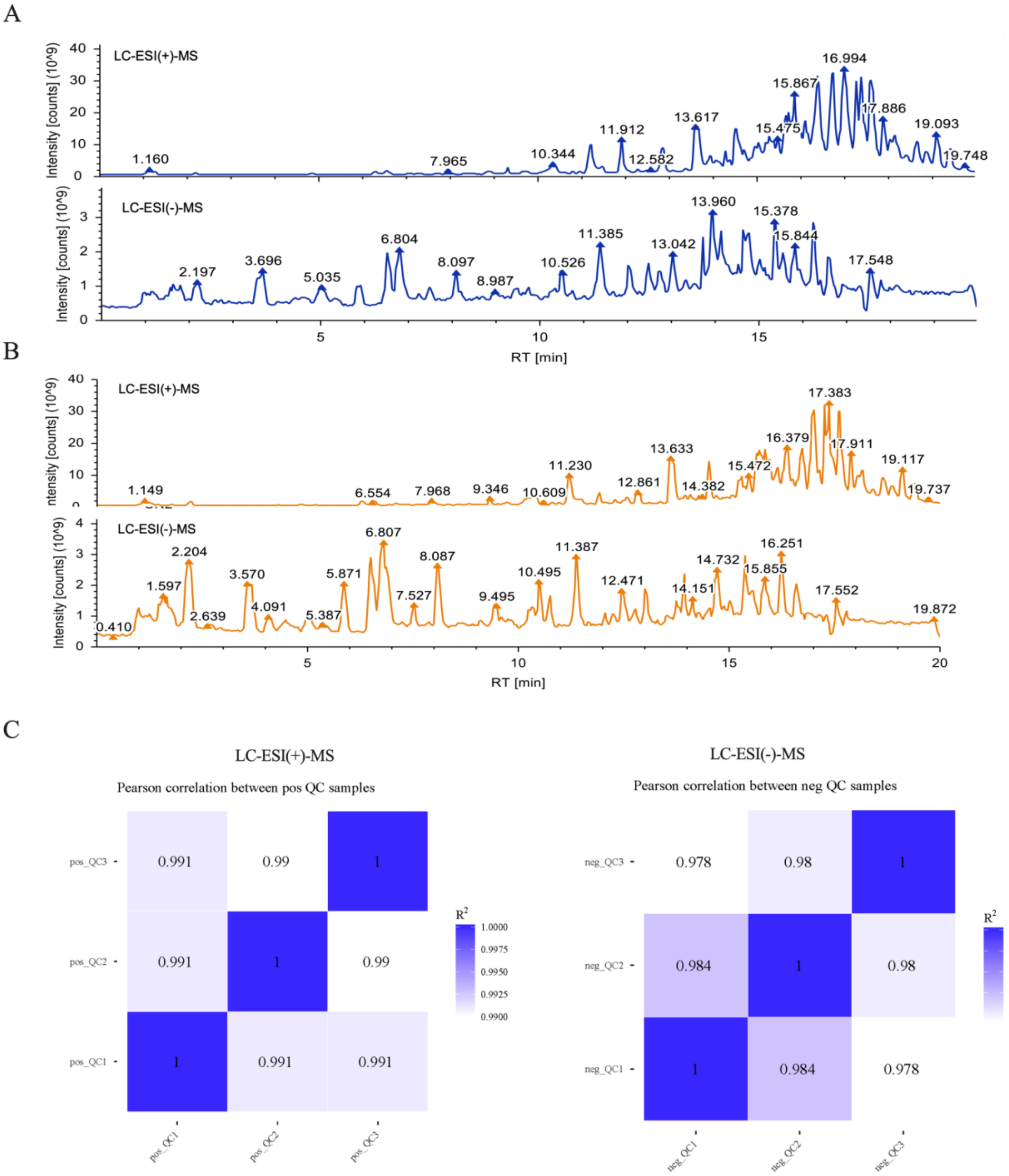

SUPPLEMENTARY FIGURE S1. The total ion chromatogram (TIC) of Black lacquer oil (BLO) and White lacquer oil (WLO) by UPLC-MS (A and B) and Correlation analysis of quality control (QC) samples (C) by UPLC-MS in lipidomic study.

3 Supplementary Table S2

SUPPLEMENTARY TABLE S2 Significantly upregulated and downregulated lipids in Black lacquer oil compared with White lacquer oil.

Name	Subclass	BLO	WLO	FC	*P*-value	VIP
**Upregulated**						
DAG (20:0/20:4)	DAG	1.11E+09	3.26E+08	3.400	2.20E-11	1.2525
DAG (20:0/18:1)	DAG	2.53E+08	8.37E+07	3.019	1.11E-08	1.2428
PA (20:4/20:4)	PA	1.98E+06	7.11E+05	2.786	1.16E-08	1.2445
Cer-NS (d18:2/16:0)	Cer	1.22E+08	1.68E+07	7.261	1.55E-08	1.2503
Cer-NP (t18:1/18:1)	Cer	1.21E+08	9.97E+06	12.164	1.88E-08	1.2395
DGTS (16:0/16:1)	DGTS	8.43E+06	2.63E+06	3.208	3.01E-08	1.2331
TAG (12:0/16:0/16:0)	TAG	4.52E+09	1.19E+09	3.811	5.98E-08	1.2423
Cer-NS (d18:2/16:1)	Cer	5.31E+07	1.70E+07	3.123	6.01E-08	1.2295
DAG (16:1/22:0/0:0)	DAG	5.42E+07	2.13E+07	2.543	6.42E-08	1.2277
SM (d29:3/12:0)	SM	6.12E+07	8.80E+06	6.952	1.08E-07	1.2385
Cer-BS (d17:2/16:0)	Cer	2.27E+07	1.18E+06	19.230	1.11E-07	1.2395
PG (6:0/26:0)	PG	6.72E+06	1.27E+06	5.312	1.27E-07	1.2227
Cer-EOS (d26:1/15:0-O-18:1)	Cer	4.74E+06	1.09E+06	4.341	1.51E-07	1.2368
LDGTS 16:0	LDGTS	1.89E+07	4.27E+06	4.416	1.87E-07	1.2206
Cer-ADS (d17:0/15:0)	Cer	4.68E+07	1.27E+07	3.677	2.49E-07	1.2416
TAG (12:0/14:0/18:0)	TAG	7.76E+08	2.33E+08	3.337	2.65E-07	1.2284
DGTS (18:1/18:2)	DGTS	2.50E+07	4.27E+06	5.868	2.81E-07	1.2311
Cer-NP (t18:1/18:0)	Cer	2.76E+07	6.32E+06	4.358	2.85E-07	1.2187
TAG (18:1/20:0/22:6)	TAG	5.77E+07	2.39E+07	2.413	2.93E-07	1.2185
LDGTS 18:1	LDGTS	2.09E+07	4.66E+06	4.480	3.12E-07	1.2208
DGTS (18:2/18:2)	DGTS	2.36E+07	6.81E+06	3.468	3.54E-07	1.2194
LPE 18:2	PE	9.28E+06	2.08E+06	4.465	1.02E-06	1.2126
Cer-NP (t18:1/16:0)	Cer	3.01E+08	4.51E+07	6.670	1.26E-06	1.2171
Cer-NS (d18:1/16:0)	Cer	2.32E+07	7.39E+06	3.143	1.69E-06	1.2095
LDGTS 18:2	LDGTS	1.44E+07	2.96E+06	4.854	2.87E-06	1.1999
TAG (15:1/19:0/19:0)	TAG	7.79E+08	2.88E+08	2.706	4.21E-06	1.2132
Cer-NP (t18:0/16:0)	Cer	1.01E+08	3.48E+07	2.895	5.88E-06	1.1924

DAG (18:1/20:4)	DAG	1.73E+07	4.33E+06	3.986	6.45E-06	1.2026
TAG (18:0/18:1/19:0)	TAG	3.45E+08	1.70E+08	2.029	7.17E-06	1.1927
FAHFA (18:1/16:0)	FAHFA	9.58E+07	4.59E+07	2.088	7.43E-06	1.1800
PA (16:0/22:1)	PA	3.25E+07	6.84E+06	4.757	7.770E-06	1.2116
TAG (14:0/16:0/16:0)	TAG	1.69E+09	5.85E+08	2.892	1.451E-05	1.1765
PE (18:1/22:6)	PE	6.82E+05	2.75E+05	2.479	2.279E-05	1.1786
DG (14:1(9Z)/16:1(9Z)/0:0)	DAG	4.26E+07	1.94E+07	2.201	2.307E-05	1.1857
Cer-EOS (d25:1/15:0-O-18:1)	Cer	1.19E+07	3.54E+06	3.354	3.033E-05	1.1864
Cer-NP (t18:0/18:0)	Cer	3.77E+07	1.73E+07	2.187	4.052E-05	1.1650
DG (18:1/20:4/0:0)[iso2]	DAG	5.02E+07	1.69E+07	2.977	5.221E-05	1.1787
DG (P-14:0/18:1)	DAG	1.68E+10	7.41E+09	2.267	6.110E-05	1.1896
Cer-AS (d18:2/16:0)	Cer	1.13E+08	5.47E+07	2.070	6.423E-05	1.1330
Cer-EOS (d24:1/15:0-O-16:0)	Cer	1.69E+07	7.28E+06	2.320	7.193E-05	1.1809
PG (18:1/18:1)	PG	2.39E+06	1.12E+06	2.126	2.510E-04	1.1239
PC (22:3e/15:0)	PC	5.08E+07	2.49E+07	2.043	2.711E-04	1.1688
PE (16:0/16:1)	PE	4.67E+06	1.92E+06	2.437	4.002E-04	1.0862
Cer-NP (t18:1/22:0)	Cer	2.69E+07	9.59E+06	2.806	4.620E-04	1.1076
SM (d15:3/28:1)	SM	1.43E+07	5.40E+06	2.645	5.374E-04	1.1359
DAG (18:1/18:1)	DAG	3.19E+09	6.83E+08	4.677	5.603E-04	1.1634
TAG (16:0/16:0/16:1)	TAG	6.79E+10	3.33E+10	2.039	6.840E-04	1.1231
TAG (18:1/18:2/20:4)	TAG	1.14E+08	1.76E+07	6.480	7.434E-04	1.1165
Cer-EOS (d19:1/15:0-O-16:0)	Cer	2.03E+07	6.25E+06	3.251	8.084E-04	1.0805
Cer-NP (t18:0/18:1)	Cer	4.21E+07	1.10E+07	3.838	8.290E-04	1.0990
DGTS (16:0/18:1)	DGTS	4.23E+07	1.95E+07	2.165	1.247E-03	1.0328
Cer-BS (d17:2/17:0)	Cer	2.78E+07	1.38E+07	2.020	1.393E-03	1.0216
DG (16:1/18:3/0:0)	DAG	1.39E+08	4.77E+07	2.911	2.830E-03	1.0165
**Downregulated**						
TAG (18:2/20:1)	TAG	5.44E+08	4.45E+09	0.122	3.24E-11	1.2549
TAG (12:0/16:2/18:2)	TAG	2.73E+07	2.34E+08	0.116	1.85E-08	1.2545
TAG (17:2/18:2/18:2)	TAG	2.43E+08	2.22E+09	0.110	1.94E-10	1.2530
TAG (18:2/18:2/22:0)	TAG	5.49E+08	3.95E+09	0.139	2.25E-11	1.2528
Cer-BDS (d22:0/16:1)	Cer	2.09E+06	1.23E+08	0.017	2.64E-08	1.2528

TAG (12:0/16:1/16:2)	TAG	2.15E+07	2.10E+08	0.102	5.08E-09	1.2526
TAG (15:1/18:1/18:2)	TAG	4.65E+08	2.76E+09	0.169	2.25E-11	1.2525
TAG (15:0/17:0/19:1)	TAG	1.20E+09	6.56E+09	0.183	1.45E-09	1.2517
TAG (15:0/18:2/18:2)	TAG	7.21E+07	4.53E+08	0.159	1.84E-10	1.2508
Cer-NP (t18:0/22:1)	Cer	4.69E+07	5.87E+08	0.080	9.47E-11	1.2498
TAG (16:1/16:2/16:3)	TAG	8.90E+06	5.50E+07	0.162	6.19E-09	1.2497
Cer-NP (t18:0/20:1)	Cer	2.87E+07	3.75E+08	0.077	3.56E-08	1.2493
TAG (18:2/18:3/19:1)	TAG	7.84E+07	7.01E+08	0.112	2.62E-10	1.2489
PE (16:0/22:1)	PE	1.69E+06	1.85E+07	0.091	2.11E-10	1.2484
TAG (14:0/18:2/18:3)	TAG	1.74E+08	9.33E+08	0.187	1.01E-08	1.2476
PE (20:0/18:2)	PE	1.70E+06	2.64E+07	0.064	7.08E-08	1.2461
TAG (12:1/14:1/16:0)	TAG	2.91E+07	1.61E+08	0.181	2.98E-07	1.2460
TAG (16:1/16:1/16:3)	TAG	3.79E+07	2.37E+08	0.160	8.11E-10	1.2458
TAG (17:1/18:2/18:2)	TAG	5.94E+08	4.00E+09	0.149	9.97E-07	1.2454
TAG (17:0/18:1/18:2)	TAG	1.01E+08	5.72E+08	0.177	2.52E-09	1.2449
TAG (17:1/18:1/18:2)	TAG	1.23E+09	6.49E+09	0.190	3.73E-07	1.2441
TAG (16:3/16:3/18:2)	TAG	2.66E+06	5.27E+07	0.050	7.07E-07	1.2440
TAG (16:0/18:2/18:5)	TAG	4.44E+07	4.02E+08	0.110	6.19E-08	1.2432
PC (19:0/19:0)	PC	6.68E+05	5.28E+06	0.126	3.39E-06	1.2432
TAG (12:0/16:1/18:2)	TAG	1.55E+07	1.17E+08	0.133	5.24E-09	1.2425
TAG (16:1/18:0/20:0)	TAG	4.50E+08	3.46E+09	0.130	2.70E-09	1.2422
TAG (12:0/18:2/18:2)	TAG	2.80E+07	2.15E+08	0.130	1.95E-07	1.2421
FAHFA (16:0/15:0)	FAHFA	1.12E+07	6.07E+07	0.185	1.06E-08	1.2408
SM (d18:1/14:1(9Z)(OH))	SM	4.23E+07	3.20E+08	0.132	6.52E-09	1.2396
TAG (18:2/20:2/22:0)	TAG	3.15E+08	2.12E+09	0.149	1.50E-08	1.2394
TAG (16:1-16:3-16:3)	TAG	4.59E+06	5.84E+07	0.079	4.57E-07	1.2364
TAG (18:2-18:2-19:1)	TAG	2.19E+08	1.47E+09	0.15	1.18E-07	1.2347
PC (22:4e/18:3)	PC	1.29E+06	1.02E+07	0.13	4.36E-07	1.2327
TAG (12:0-16:3-18:2)	TAG	6.88E+06	7.12E+07	0.10	8.87E-06	1.2322
PE (18:0/20:0)	PE	4.86E+06	5.53E+07	0.09	1.59E-05	1.2308
TAG (16:2/16:3/18:2)	TAG	1.12E+07	1.11E+08	0.10	1.82E-05	1.2294
PC (16:0/18:2)	PC	2.67E+06	5.06E+07	0.05	1.47E-06	1.2271

Cer-BDS (d23:0/19:1)	Cer	6.25E+07	5.06E+08	0.12	7.65E-06	1.2270
TAG (18:1/18:2/19:1)	TAG	1.27E+08	8.99E+08	0.14	1.28E-05	1.2210
TAG (18:2/20:1/22:0)	TAG	1.56E+08	7.90E+08	0.20	2.56E-07	1.2184
TAG (18:2/18:2/18:3)	TAG	6.38E+08	3.30E+09	0.19	4.05E-05	1.2142
SM(d18:0/22:3)	SM	3.30E+07	2.08E+08	0.16	1.67E-05	1.2129
TAG (16:0/18:1/22:4)	TAG	1.92E+07	1.17E+08	0.16	6.66E-06	1.2066
PEtOH (25:0/18:2)	PEtOH	2.43E+06	3.03E+07	0.08	4.36E-05	1.2048
PC (16:2e/27:0)	PC	1.53E+07	8.41E+07	0.18	1.77E-06	1.2046
Cer-AS (d18:2/24:0)	Cer	4.32E+07	5.61E+08	0.08	3.40E-06	1.1952
PEtOH (24:0/18:2)	PEtOH	2.35E+06	1.39E+07	0.17	5.79E-05	1.1844
TAG (18:2/18:2/20:0)	TAG	1.29E+09	9.75E+09	0.13	7.25E-06	1.1798
Cer-AS (d16:1/25:0)	Cer	5.98E+06	7.29E+07	0.08	2.22E-05	1.1757
Cer-NP (t18:0/23:1)	Cer	2.33E+07	2.53E+08	0.09	8.66E-06	1.1754
Cer-NP (t27:1/16:0)	Cer	7.59E+06	4.25E+07	0.18	4.34E-05	1.1548
PE (18:0/18:1)	PE	2.82E+06	3.14E+07	0.09	1.15E-04	1.1488
Cer-NP (t18:0/21:1)	Cer	6.66E+06	4.06E+07	0.16	2.57E-04	1.0914
Cer-AS (d18:2/22:0)	Cer	8.97E+07	5.50E+08	0.16	3.32E-04	1.0882
Cer-NS (d18:2/23:0)	Cer	3.99E+06	2.37E+07	0.17	3.64E-04	1.0807
Cer-AP (t20:0/24:0)	Cer	2.69E+06	1.94E+07	0.14	2.04E-03	1.0199

BLO: mean value of Black lacquer oil, WLO: mean value of White lacquer oil, FC: Fold Change, VIP: Variable Importance in the Projection.

4 Supplementary Figure S2

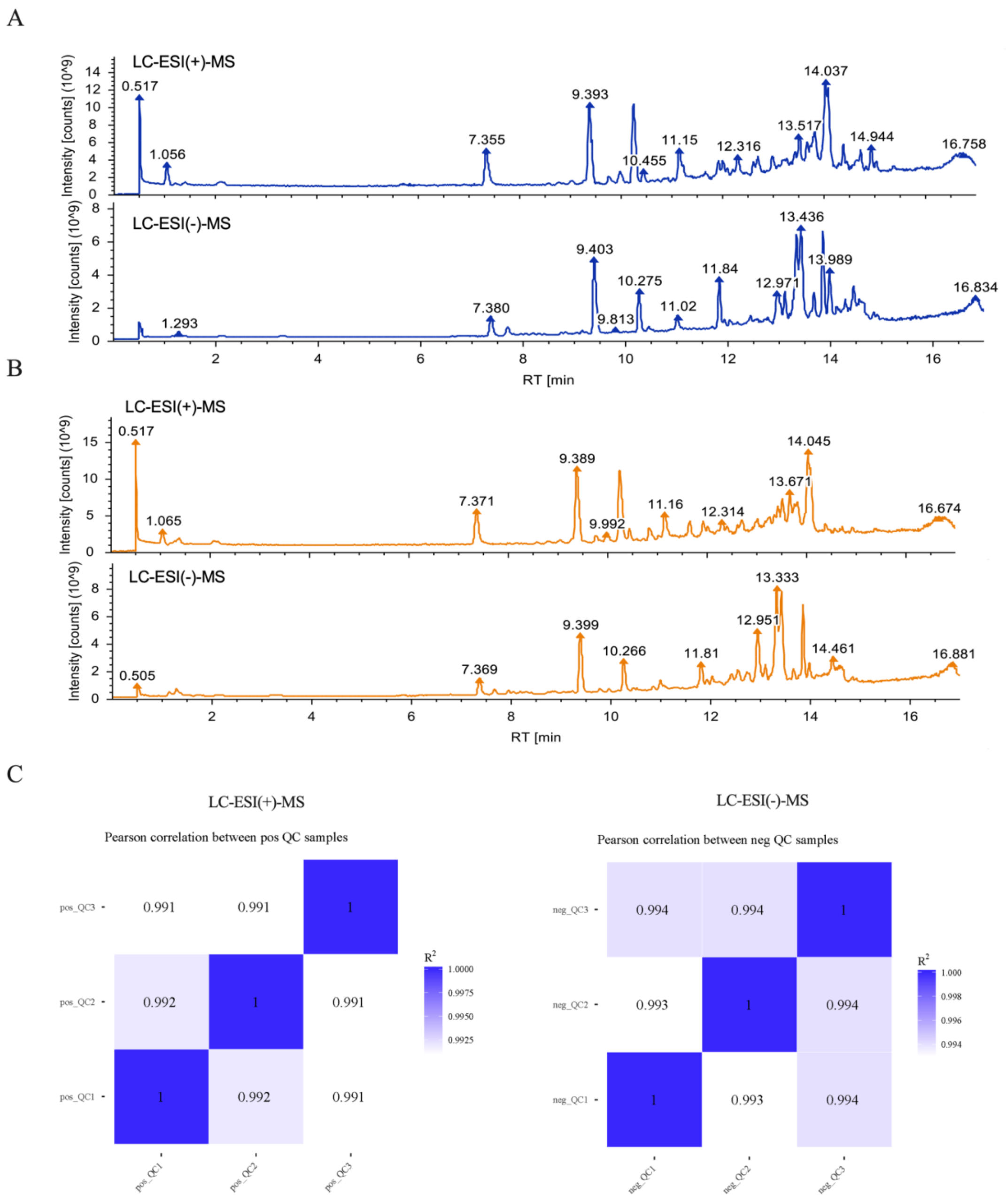

SUPPLEMENTARY FIGURE S2. The total ion chromatogram (TIC) of Black lacquer oil (BLO) and White lacquer oil (WLO) by UPLC-MS (A and B) and Correlation analysis of quality control (QC) samples (C) by UPLC-MS in untargeted metabolomics study.

5. Supplementary Table S3

SUPPLEMENTARY TABLE S3 Differential metabolites in black lacquer oil compared to white lacquer oil

Name	Subclass	BLO	WLO	FC	*P*-value	VIP
Isocitric acid	Organic acid	3.35E+08	1.33E+07	25.237	1.11E-14	1.222
Oxypeucedanin	Coumarin	2.35E+07	2.02E+06	11.656	4.15E-14	1.201
3-Furoic acid	Organic acid	2.12E+07	2.64E+06	8.026	1.23E-13	1.238
L-Iditol	Sugar	2.40E+08	1.85E+07	13.003	6.19E-13	1.215
Benzoylhypacoitine	Alkaloid	1.68E+08	5.14E+06	32.686	6.61E-13	1.206
Isopropyl beta-D-glucopyranoside	Sugar	1.42E+07	1.44E+06	9.905	8.24E-12	1.216
DL-Stachydrine	Alkaloid	9.94E+08	1.29E+08	7.683	8.71E-12	1.216
Stachyose	Sugar	1.40E+07	1.61E+06	8.736	1.04E-11	1.225
D-Arabitol	Sugar	1.16E+07	1.10E+06	10.509	1.36E-11	1.231
Lipoamide	Coenzyme	5.63E+06	8.86E+05	6.354	1.38E-11	1.233
5-Methoxyindole-3-acetic acid	Organic acid	1.01E+07	1.72E+06	5.882	1.56E-11	1.227
trans-Aconitic acid	Organic acid	1.35E+08	5.11E+06	26.404	1.88E-11	1.214
Citronellyl acetate	Terpenoid	2.68E+07	2.58E+06	10.365	2.11E-11	1.236
Ethyl ferulate	Phenylpropanoid	7.75E+07	7.87E+06	9.852	2.64E-11	1.219
L-Threonic acid	Sugar	1.21E+07	1.86E+06	6.503	4.03E-11	1.244
DL-Malic acid	Organic acid	5.20E+08	9.33E+07	5.566	4.04E-11	1.239
(+)-Catechin	Polyphenol	1.63E+07	1.94E+06	8.398	1.16E-10	1.219
4-Guanidinobutyric acid	lipid	7.28 E+07	1.17 E+07	6.24	7.20E-09	1.22
1,2,3,7-Tetramethoxyxanthone	Xanthone	3.56E+07	2.67E+06	13.322	1.69E-10	1.217
α-Lactose	Sugar	3.17E+07	6.22E+06	5.102	2.47E-10	1.228
4-Hexyloxyaniline	Aniline	2.37E+07	3.71E+06	6.382	2.58E-10	1.215
Angeloyl-(+)-gomisin K3	Lignan	4.56E+06	6.39E+05	7.130	3.73E-10	1.224
Dulcitol	Sugar	2.32E+08	1.69E+07	13.741	3.97E-10	1.226
Isopimpinellin	Coumarin	3.70E+06	5.50E+05	6.722	3.99E-10	1.240
Betaine	Alkaloid	8.55E+08	1.53E+08	5.577	4.64E-10	1.216
Trigonelline	Alkaloid	5.19E+07	9.36E+06	5.543	6.08E-10	1.198
D-Gluconic acid	Sugar	1.39E+07	1.80E+06	7.738	8.33E-10	1.215
Danshenol C	Terpenoid	5.23E+06	8.88E+05	5.892	1.25E-09	1.230

pentane-1,2,3,4,5-pentol	Sugar	9.78E+07	1.28E+07	7.625	2.30E-09	1.214
Maltopentaose	Sugar	1.31E+06	1.99E+05	6.567	2.85E-09	1.224
Uridine monophosphate (UMP)	Nucleoside	1.55E+06	1.10E+05	14.125	3.20E-09	1.224
N-Acetyl-Dl-glutamic acid	Amino acid	7.89E+06	7.57E+05	10.423	3.23E-09	1.193
D-Raffinose	Sugar	1.70E+07	1.87E+06	9.117	4.21E-09	1.213
Hypoxanthine-9-beta-D-Arabinofuranoside	Sugar	3.43E+06	6.35E+05	5.398	4.91E-09	1.234
Myristicin	Phenylpropanoid	5.08E+08	1.85E+07	27.457	6.11E-09	1.206
4-Guanidinobutyric acid	Alkaloid	7.28E+07	1.17E+07	6.236	7.20E-09	1.219
Thioctic acid	Coenzyme	2.74E+06	4.77E+05	5.731	9.93E-09	1.236
Nortrachelogenin	Lignan	6.76E+06	1.15E+06	5.858	1.36E-08	1.249
Dehydrodiisoeugenol	Lignan	8.35E+06	3.31E+05	25.276	1.82E-08	1.207
Praeruptorin A	Coumarin	2.50E+07	2.82E+06	8.861	2.26E-08	1.219
Gluconolactone	Sugar	4.54E+06	7.47E+05	6.074	3.67E-08	1.217
Theophylline-7-acetic acid	Alkaloid	1.69E+06	2.74E+05	6.192	5.98E-08	1.221
Palmitic Acid	Lipid	1.14E+08	2.27E+07	5.010	6.20E-08	1.213
Corticosterone	Hormone	1.05E+07	1.73E+06	6.084	1.36E-07	1.177
6-Pentyl-2H-pyran-2-one	Lactone	2.22E+08	3.70E+07	6.003	1.99E-07	1.206
Gluconic acid	Sugar	3.68E+07	6.26E+06	5.879	2.37E-07	1.193
Fargesin	Lignan	1.26E+07	2.24E+06	5.637	3.41E-07	1.222
Maltotetraose	Sugar	4.42E+06	6.00E+05	7.366	5.31E-07	1.220
Phloretin	Flavonoid	1.64E+06	2.67E+05	6.138	1.87E-06	1.219
Flavin mononucleotide (FMN)	Nucleoside	1.27E+07	2.25E+06	5.641	3.54E-06	1.214
UDP-N-acetylglucosamine	Sugar	6.82E+05	1.02E+05	6.718	4.90E-06	1.204
Picrotoxinin	Terpenoid	3.73E+06	6.25E+05	5.967	5.73E-06	1.179
Hesperetin	Flavonoid	1.90E+06	3.38E+05	5.626	6.22E-06	1.202
19(R)-hydroxy Prostaglandin E2	Hormone	3.42E+06	4.64E+05	7.358	1.35E-05	1.125
7-Methylxanthine	Nucleoside	1.36E+07	1.98E+06	6.857	1.56E-05	1.188
Solanidine	Alkaloid	7.57E+06	8.56E+05	8.852	1.58E-05	1.195
D-(+)-Maltose	Sugar	4.44E+07	7.38E+06	6.021	1.63E-05	1.199
